# Fluorescent-target imaging and real-time beam diagnostics at the High Energy Photon Source

**DOI:** 10.1107/S1600577526004984

**Published:** 2026-06-16

**Authors:** Qun Zhang, Yao Zhao, Yu Liu, Zhanxin Gu, Sai Liu, Qihui Duan, Gang Li, Yi Zhang, Chenglong Zhang

**Affiliations:** a Institute of High Energy Physics Chinese Academy of Sciences Beijing100049 People’s Republic of China; b High Energy Photon Source (HEPS), Beijing Synchrotron Radiation Facility (BSRF), Beijing100049, People’s Republic of China; c Beijing Key Laboratory on Integration and Analysis of Large-Scale Stream Data North China University of Technology Beijing100144 People’s Republic of China; ESRF – The European Synchrotron, France

**Keywords:** fourth-generation synchrotron radiation, beam diagnostics, fluorescent-target imaging, *EPICS*, *Mamba Data Worker*, high-throughput data management

## Abstract

Fourth-generation synchrotron radiation sources based on diffraction-limited storage rings impose stringent requirements on beamline alignment precision, diagnostic reliability, and real-time monitoring under high-brightness operating conditions. To address these requirements, an integrated fluorescent-target imaging and real-time beam diagnostic platform has been developed at the High Energy Photon Source (HEPS). The system incorporates a radiation-tolerant and ultra-high-vacuum-compatible mechanical assembly with diamond and YAG:Ce scintillators for different heat-load conditions. A distributed multi-camera architecture implemented within the *Experimental Physics and Industrial Control System* (*EPICS*) *areaDetector* framework enables synchronized multi-angle monitoring of beam profiles. Key beam parameters are extracted through online image processing and published as *EPICS* process variables for control-system integration. To support high-throughput diagnostic data handling, the platform further integrates the *Mamba Data Worker* framework, enabling coordinated multi-target acquisition, HDF5 storage, and automated metadata ingestion into a dedicated HEPS beamline alignment database. Representative deployment demonstrated stable synchronized operation of 13 cameras, with online analysis completed in less than 20 ms and end-to-end latency below 50 ms. These results establish a practical and scalable framework for beamline diagnostics, alignment support, and data-driven optimization at HEPS.

## Abstract 2

A fluorescent-target imaging and real-time beam diagnostic platform has been developed for the High Energy Photon Source (HEPS), integrating synchronized multi-camera acquisition within the *Experimental Physics and Industrial Control System* (*EPICS*) *areaDetector* framework, online beam-parameter extraction, and *Mamba Data Worker* enabled data management. The platform demonstrated stable 13-camera operation with sub-20 ms online analysis and end-to-end latency below 50 ms, supporting scalable beamline alignment, diagnostics, and future data-driven optimization at fourth-generation synchrotron facilities.

## 1. Introduction

Fourth-generation synchrotron radiation facilities employing diffraction-limited storage rings have significantly reduced natural emittance, leading to major improvements in photon-beam brightness and transverse coherence. Representative projects such as the Extremely Brilliant Source at the European Synchrotron Radiation Facility (ESRF–EBS), the Advanced Photon Source Upgrade (APS-U), and the High Energy Photon Source (HEPS) exemplify the rapid development of this class of light source and the expanding experimental capabilities in high-resolution imaging, coherent scattering, and time-resolved studies (Raimondi *et al.*, 2023 ▸; Jiao *et al.*, 2018 ▸; Hettel, 2021 ▸). At the same time, higher brightness, smaller beam sizes, and increased flux density place more stringent requirements on beamline alignment, online beam characterization, and diagnostic reliability.

Under these conditions, robust beam diagnostic methods are essential for both beamline commissioning and routine operation. Among the available approaches, fluorescent-target imaging remains one of the most practical and widely used techniques because it provides direct two-dimensional visualization of beam position, profile, and intensity distribution. In this method, incident X-rays excite a scintillator material and generate visible-light emission, which is then collected by optical components and recorded by an imaging camera. The performance of scintillator-based indirect X-ray imaging systems has been extensively investigated, particularly with respect to light yield, spatial resolution, temporal response, and radiation tolerance under synchrotron irradiation (Rutherford *et al.*, 2016 ▸). In addition, YAG:Ce, one of the commonly used scintillator materials in beam imaging, has attracted continued interest for the optimization of its scintillation properties (Zapadlík *et al.*, 2022 ▸).

At advanced synchrotron radiation facilities, imaging-based beam diagnostics remain important for beam observation, alignment, and quantitative beam characterization. For example, imaging-based diagnostics at ESRF have been used to monitor beam shape and beam position under high-power-density conditions, highlighting the continuing importance of screen-based beam observation in modern synchrotron beamlines (van Silfhout *et al.*, 2020 ▸). However, as beamlines evolve toward higher brightness, smaller beam sizes, and increasingly distributed diagnostic layouts, conventional locally deployed imaging devices are often insufficient to support synchronized multi-point observation, standardized control, and online quantitative analysis. These developments motivate the construction of integrated diagnostic platforms that combine robust target assemblies, coordinated image acquisition, real-time parameter extraction, and scalable data handling. In this context, the *Experimental Physics and Industrial Control System* (*EPICS*) has become a widely adopted distributed architecture for instrumentation control and data exchange in large scientific facilities (Dalesio *et al.*, 1994 ▸), while the *areaDetector* framework provides a flexible interface for integrating two-dimensional detectors and industrial cameras into unified imaging pipelines (Rivers, 2010 ▸).

At HEPS, related software infrastructures have been developed to support beamline experiments and high-throughput data handling. *Mamba* provides a systematic software solution for experiment organization and execution at HEPS (Liu *et al.*, 2022 ▸), while a high-throughput big-data orchestration and processing system supports coordinated acquisition and downstream processing of large experimental data flows (Li *et al.*, 2023 ▸). Building on these developments and motivated by the diagnostic requirements of fourth-generation synchrotron beamlines, we present an integrated fluorescent-target imaging and beam diagnostics system for HEPS. The system combines a radiation-tolerant target assembly suitable for vacuum and high-flux environments, synchronized multi-camera acquisition implemented within the *EPICSareaDetector* framework, real-time extraction of key beam parameters, and a data orchestration layer for coordinated processing and storage. The following sections describe the mechanical design, synchronized imaging architecture, real-time analysis strategy, and high-throughput data-management workflow of the proposed system.

## 2. Mechanical design of fluorescent targets for HEPS beamline diagnostics

**Figure 1:**
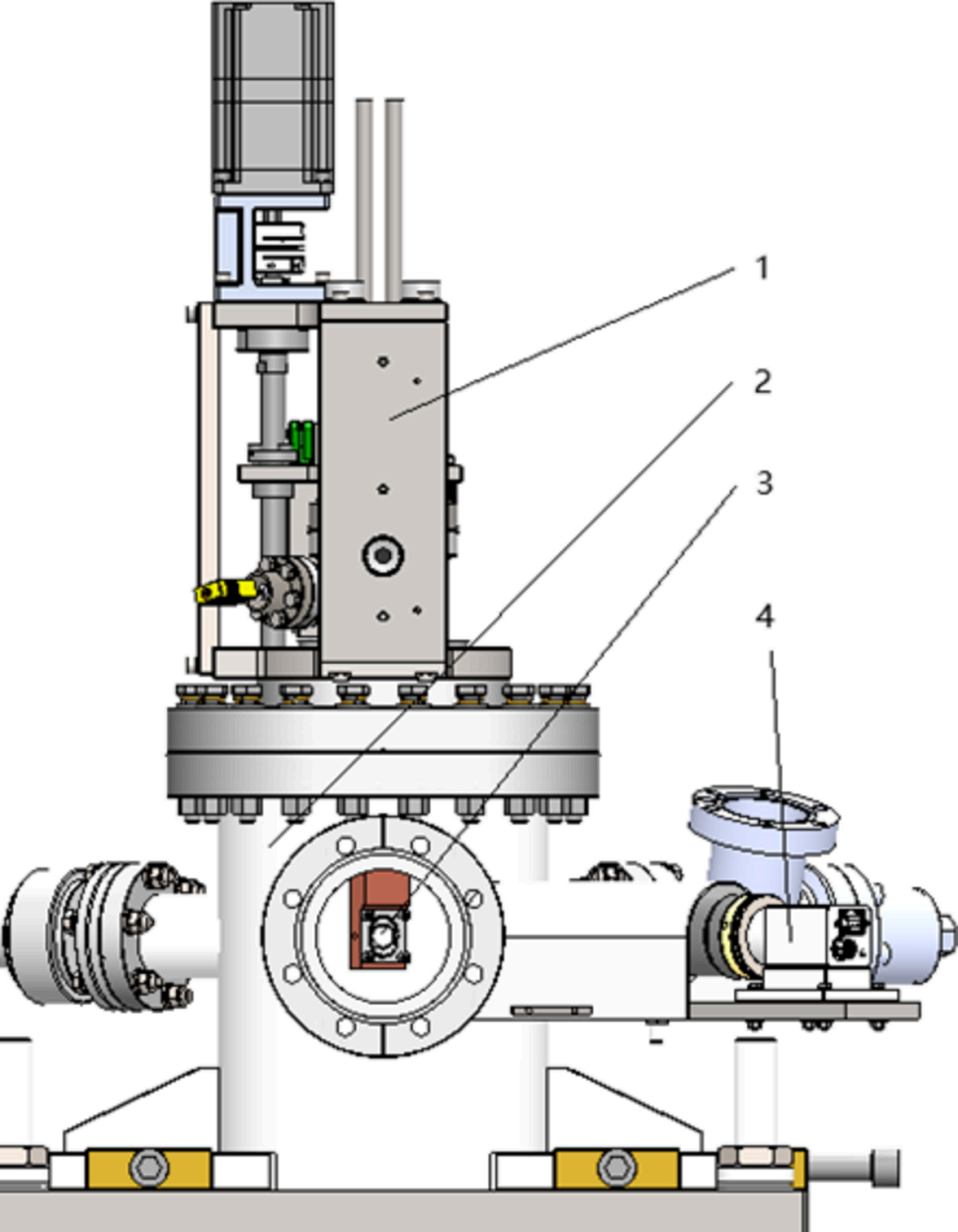
Structural schematic of the fluorescent target system, highlighting subsystems (1) drive assembly, (2) vacuum chamber, (3) target holder, and (4) imaging optics. In a representative white-beam implementation at HEPS, a 20 mm-diameter diamond scintillator is used together with a 35 mm translation stroke and a 1× imaging system, corresponding to a representative pixel resolution of 5.86 µm per pixel.

The fluorescent-target system is a key component for beam diagnostics at HEPS beamlines. Its mechanical design must satisfy several practical requirements, including compatibility with ultra-high-vacuum (UHV) conditions, resistance to radiation and thermal load, stable and reproducible target positioning, and flexible adaptation to different beamline configurations. As shown in Fig. 1 ▸, the fluorescent-target system consists of the target assembly, motion mechanism, vacuum interface, cooling structure, and external imaging optics arranged in a modular configuration. Representative HEPS implementations adopt different scintillator and optical configurations according to beamline requirements and diagnostic tasks.

### 2.1. Mechanical architecture and motion design

The fluorescent target system consists of four principal subsystems: a drive assembly, a vacuum chamber, a target holder, and an imaging module. The vacuum chamber is fabricated to ensure reliable operation under UHV conditions, while the target holder adopts a modular configuration that facilitates rapid replacement of different scintillators according to beamline requirements. The target assembly is mounted on a motor-driven linear translation stage incorporating a precision lead-screw actuator. This motion system enables smooth insertion and retraction of the scintillator along the beam path and was designed to achieve a positioning accuracy of ±1 µm and a repeatability of ±5 µm under beamline operating conditions. The motion system is integrated into the beamline chamber through a sealed flange interface, allowing reliable vacuum isolation while supporting rapid target deployment during commissioning and routine beam diagnostics.

This structural and motion-control configuration provides the mechanical foundation for stable fluorescent-target operation across diverse HEPS beamlines. In addition to ensuring accurate positioning at the beam-interaction point, the modular arrangement also improves maintainability and adaptability for stations with different installation geometries and diagnostic demands.

### 2.2. Scintillator selection and thermal management

The selection of scintillator material is closely related to beam characteristics and diagnostic objectives. For white-beam applications, monocrystalline diamond scintillators are preferred because of their high thermal conductivity and strong resistance to radiation damage, which help maintain stable performance under intense photon-flux exposure (Martin *et al.*, 2017 ▸). For monochromatic beam diagnostics, YAG:Ce single crystals are more suitable because their high luminous efficiency and favorable scintillation properties are advantageous for image-based beam profiling, particularly when small beam spots and higher optical contrast are required (Nikl, 2006 ▸). The modular holder design enables flexible deployment of the appropriate scintillator type for different beamline stations and operating modes.

Since scintillator performance is strongly influenced by thermal load, thermal management is also a central consideration in the target design. Continuous X-ray irradiation may deposit substantial energy within the scintillator, potentially reducing emission efficiency or causing structural degradation if the generated heat is not effectively removed (Martin *et al.*, 2017 ▸; Nikl, 2006 ▸). For this reason, the target holder in white-beam applications incorporates an active liquid-cooling structure to improve heat dissipation during prolonged exposure. In monochromatic beamlines, where the incident power density is much lower, heat accumulation is generally less severe, and the scintillator can operate reliably without additional active cooling. This differentiated design improves both the robustness and the versatility of the system across different diagnostic scenarios.

### 2.3. Optical imaging configuration and beamline deployment validation

**Figure 2:**
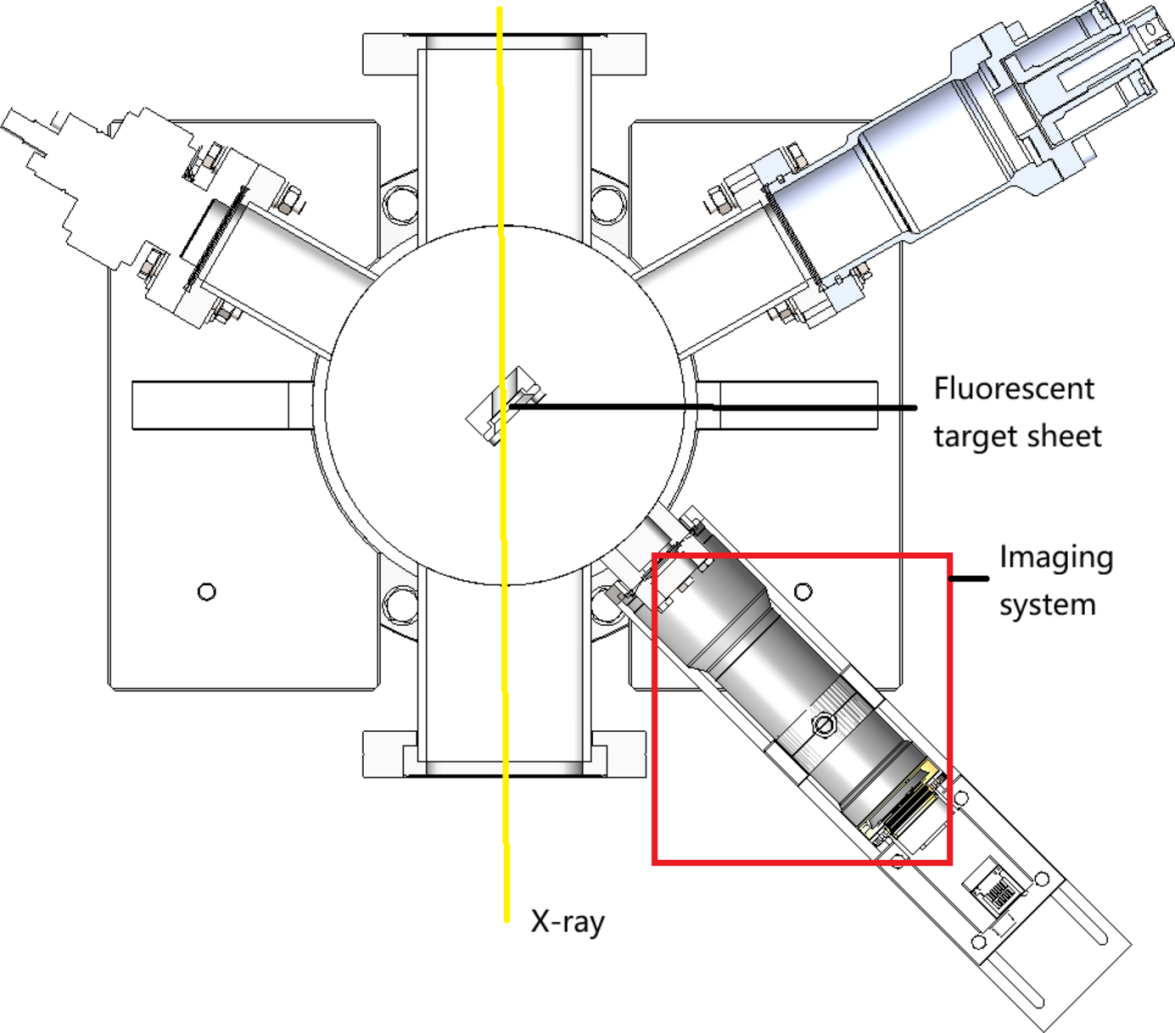
Simplified imaging principle, illustrating visible-light generation from X-ray interactions and optical path redirection via reflective components. Representative HEPS implementations include monochromatic microbeam imaging with a 20 mm-diameter YAG:Ce scintillator and switchable magnification, as well as large-field imaging using a 65 mm × 30 mm diamond target and a mirror-assisted optical path.

The imaging optics are positioned outside the vacuum chamber and observe the scintillator through an optical window, as illustrated in Fig. 2 ▸. This configuration isolates the camera and lens system from the beamline vacuum environment while preserving direct optical access to the visible-light signal generated by X-ray excitation of the scintillator. The optical system can operate in selectable high-magnification or low-magnification modes according to different beam sizes and diagnostic tasks. The low-magnification mode is suitable for rapid observation of beam position and overall beam profile during alignment and commissioning, whereas the high-magnification mode is used when finer spatial details of the beam image are required. For beamlines with constrained installation space or large chamber geometries, reflective optical pathways can be introduced to redirect scintillation light toward the imaging system while maintaining effective image collection.

The integrated mechanical-optical design was validated through representative deployment and repeated operation under beamline conditions relevant to the first batch of HEPS beamlines. To cover the major application scenarios, verification was carried out for both high-heat-load and conventional monochromatic configurations, including beamlines for imaging, scattering, and related diagnostic applications. In high-heat-load cases such as the Hard X-Ray Imaging beamline (ID21), the diamond-target configuration with active cooling maintained stable operation during continuous exposure, and no obvious thermally induced image degradation was observed over 2–4 h of routine beam observation. For conventional monochromatic configurations represented by the Microfocusing X-ray Protein Crystallography beamline (ID02), Hard X-ray Coherent Scattering beamline (ID09), and Hard X-ray Nanoprobe Multimodal Imaging beamline (ID19), the YAG:Ce-based target assembly operated reliably without additional active cooling and provided fluorescent images suitable for beam-position identification and beam-profile analysis. Repeated insertion and retraction tests over more than 50 operation cycles confirmed stable motion of the target assembly and reproducible deployment of the scintillator at the beam-interaction position, with experimental positioning repeatability maintained within ±5 µm. In addition, the modular structure facilitated rapid target replacement and adaptation to beamlines with different spatial layouts and optical-path constraints, including stations requiring reflective optical redirection. These results demonstrate that the proposed mechanical and optical design provides a practical and adaptable platform for fluorescent-target-based beam diagnostics across the principal application scenarios of the first-batch HEPS beamlines.

## 3. Multi-camera synchronization and real-time analysis

Building upon the validated mechanical-optical fluorescent-target platform described above, a synchronized multi-camera imaging system was implemented for HEPS beamlines to support reliable beam diagnostics under high-brightness operating conditions. Compared with conventional single-camera arrangements, the multi-camera configuration improves diagnostic robustness through concurrent monitoring of fluorescent targets from multiple viewing positions and is particularly advantageous for beamlines with constrained installation space or complex optical layouts.

### 3.1. Synchronized multi-camera architecture

**Figure 3:**
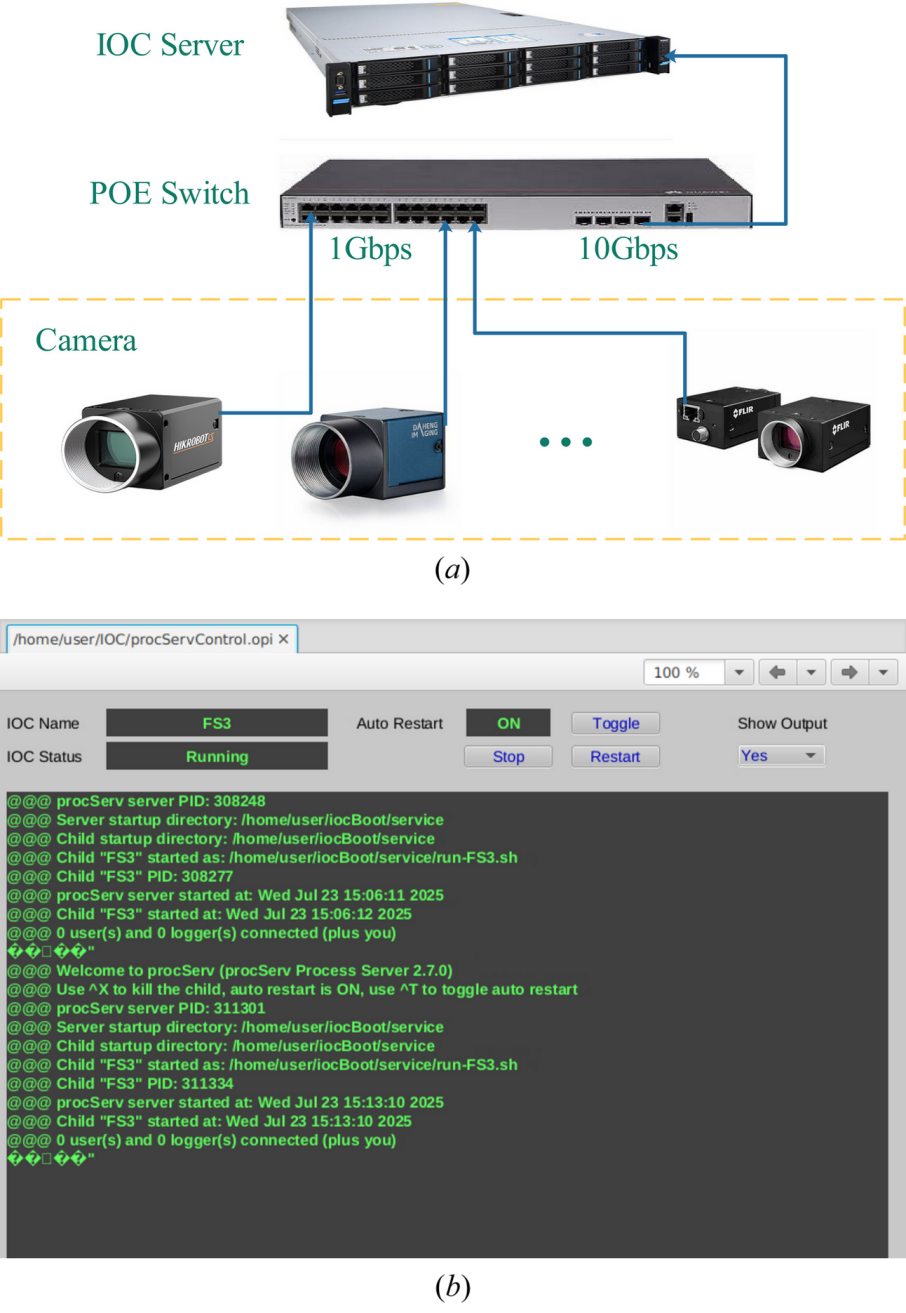
System architecture for synchronized multi-camera beam diagnostics at HEPS. (*a*) Networking topology and camera synchronization architecture. (*b*) Distributed IOC supervision and process management.

To support coordinated beam observation across distributed beamline positions, a synchronized multi-camera architecture was established within the *EPICS* framework. The imaging system is constructed using industrial cameras compliant with the GigE Vision and GenICam standards, ensuring high-throughput image transmission and protocol-level interoperability within the control environment (Automated Imaging Association, 2018 ▸; European Machine Vision Association, 2019 ▸). As shown in Fig. 3 ▸(*a*), multiple cameras are connected through a dedicated Ethernet infrastructure to an IOC server, forming a distributed imaging network for synchronized acquisition. Each camera operates as an independent acquisition node governed by a dedicated IOC, which is responsible for camera configuration, image capture, and data dissemination.

To improve reliability during continuous operation, IOC processes are isolated and supervised using procServ-based process management (Lange & Keer, 2010 ▸), enabling controlled initialization, real-time supervision, and fault recovery, as illustrated in Fig. 3 ▸(*b*). This design supports scalable expansion of the imaging system while maintaining operational stability and preventing single-point failures from propagating across the camera array. Representative deployment at the Structural Dynamics beamline (ID23) demonstrated synchronized operation of 13 cameras within the same control framework.

### 3.2. *EPICS*-based online processing and parameter extraction

**Figure 4:**
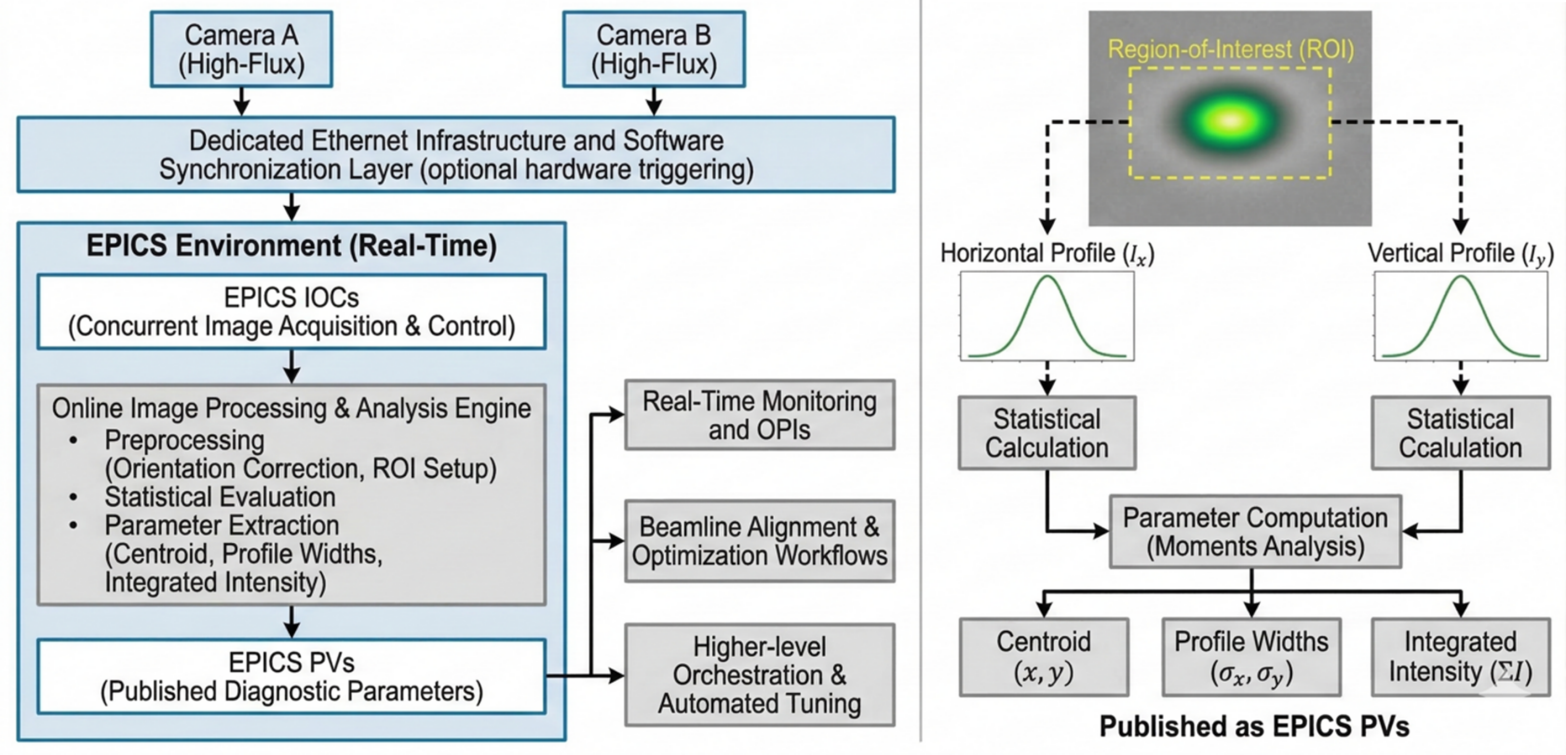
Integrated *EPICS*-based acquisition (left) and real-time parameter extraction workflow (right).

Within this synchronized imaging framework, real-time image-based diagnostics are implemented by integrating online image processing and parameter extraction directly into the *EPICS* environment. The overall workflow is summarized in Fig. 4 ▸. After image acquisition, the data undergo basic preprocessing, including orientation correction and region-of-interest selection, followed by statistical evaluation for quantitative beam characterization.

The extracted parameters include beam centroid position, profile widths in the horizontal and vertical directions, and integrated intensity. These quantities are calculated from image moments and profile statistics and then published as *EPICS* process variables for beamline monitoring and control. This design enables immediate access to image-derived beam information through a unified software environment, while allowing configuration updates and acquisition control to be propagated consistently across distributed cameras. In this way, the system not only supports synchronized image capture, but also establishes a standardized pathway from raw image streams to online numerical diagnostics.

### 3.3. Representative outputs and performance validation

**Figure 5:**
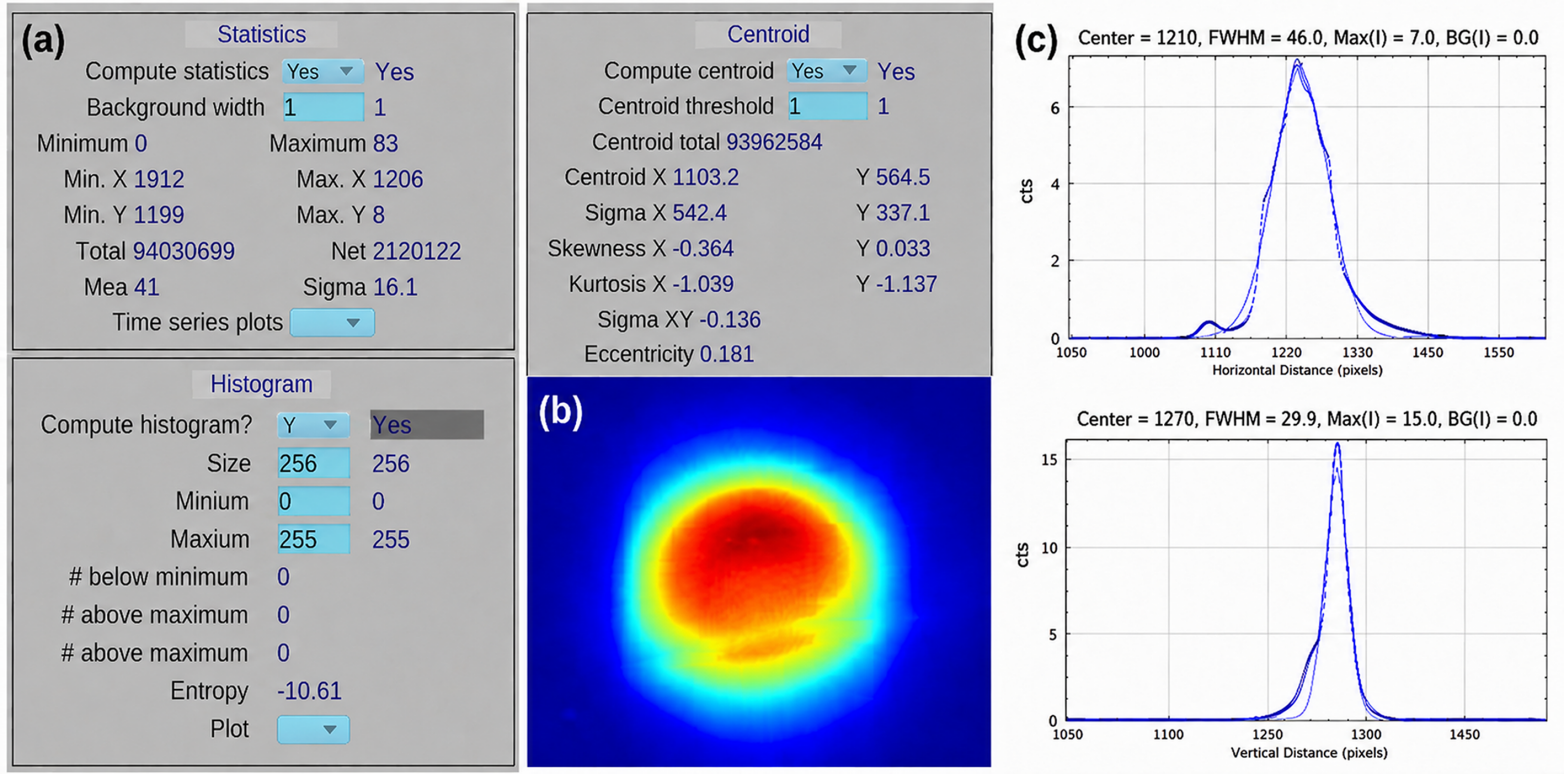
Representative online diagnostic outputs and quantitative beam analysis. (*a*) Online statistical, centroid-based, and histogram-derived parameter output in the *EPICS* environment. (*b*) Representative pseudocolor beam image acquired from the fluorescent target. (*c*) ROI-based horizontal and vertical profile extraction for quantitative evaluation of beam position and beam width.

Representative online outputs and quantitative beam-analysis results are shown in Fig. 5 ▸. The online parameter interface provides real-time display of statistical quantities, centroid coordinates, and histogram-based descriptors, enabling rapid observation of beam-position and profile variations during optical adjustment and routine beamline operation. In parallel, the fluorescent-target image provides clear visualization of the beam footprint, while ROI-based horizontal and vertical profile extraction enables quantitative evaluation of beam-center position and beam width.

In routine operation, the synchronized multi-camera architecture combined with *EPICS*-based real-time analysis provided stable beam observation and reproducible parameter extraction across different imaging positions. Representative benchmark tests further demonstrated stable high-throughput performance, where synchronized multi-camera acquisition was achieved without packet loss. IOC-side parameter extraction remained below 20 ms, and the end-to-end acquisition-to-analysis delay remained below 50 ms. These results demonstrate that the proposed system supports not only synchronized image acquisition but also quantitative online beam characterization, thereby providing a reliable technical foundation for beamline diagnostics at HEPS.

## 4. *Mamba Data Worker* for high-throughput data management in HEPS beamlines

While the *EPICS*-based multi-camera system described in Section 3 provides real-time visualization and interactive analysis capabilities, the growing scale and complexity of beamline diagnostics at HEPS demand a more robust solution for high-throughput data management. As the number of fluorescent targets increases across beamline stations, the volume of image data generated during synchronized acquisition can easily exceed the processing capacity of conventional single-node architectures. To address this challenge, the *Mamba Data Worker* (*MDW*) (Liu *et al.*, 2022 ▸; Li *et al.*, 2023 ▸) platform has been integrated into the fluorescent-target imaging workflow. *MDW* is a distributed data orchestration framework originally developed for high-speed detector data acquisition at HEPS, built upon ZeroMQ messaging and a modular worker architecture. In this section, we describe how *MDW* is applied to fluorescent target diagnostics, focusing on three key aspects: multi-target data acquisition and alignment, fan-out distribution to multiple downstream applications, and persistent storage in HDF5 format with read/write support.

### 4.1. Multi-target data acquisition

The *MDW* framework addresses multi-target data acquisition by interfacing with heterogeneous detector devices deployed along the beamline, including area detectors, industrial cameras, motors, and sensors. Each device serves as an independent data stream source and is managed by a dedicated Detector Worker process. Prior to acquisition, the control system configures experiment-specific parameters for each device, such as frame rate and exposure time, ensuring that all sources operate under consistent timing conditions. Critically, the data generation mode for each device is configured to use Single-Writer/Multiple-Reader (SWMR) access, enabling real-time writing of multi-dimensional data into HDF5 files (The HDF Group, 1997–2024 ▸).

During data acquisition, each Detector Worker continuously writes incoming frames and associated metadata into its designated HDF5 file. The HDF5 format inherently supports storage of arbitrarily high-dimensional data arrays, accommodating the diverse dimensionalities produced by different detector types—from one-dimensional sensor traces to two-dimensional camera images and multi-dimensional spectral datasets. As data accumulate in the individual HDF5 files, the *MDW* alignment mechanism initiates a coordinated readout process. The system first computes the target data block length, denoted as length, representing the number of frames to be retrieved in the current batch. If the remaining data in any file are less than the default block size, the value of length is adjusted to match the smallest available remainder, ensuring that no file is read beyond its current extent.

### 4.2. Multi-target data alignment

The alignment process monitors each HDF5 file to determine whether length frames have been written. For any file that has not yet accumulated the required amount of data, the system performs a dataset flush operation to synchronize the on-disk state with the writer’s in-memory buffer, and subsequently retrieves the newly available data (denoted as newdata). The retrieved newdata is appended to the corresponding per-file read buffer, and the actual buffer length reallen is updated accordingly as reallen = reallen + length. This incremental buffering strategy avoids the overhead of re-reading previously consumed data and maintains a minimal memory footprint.

**Figure 6:**
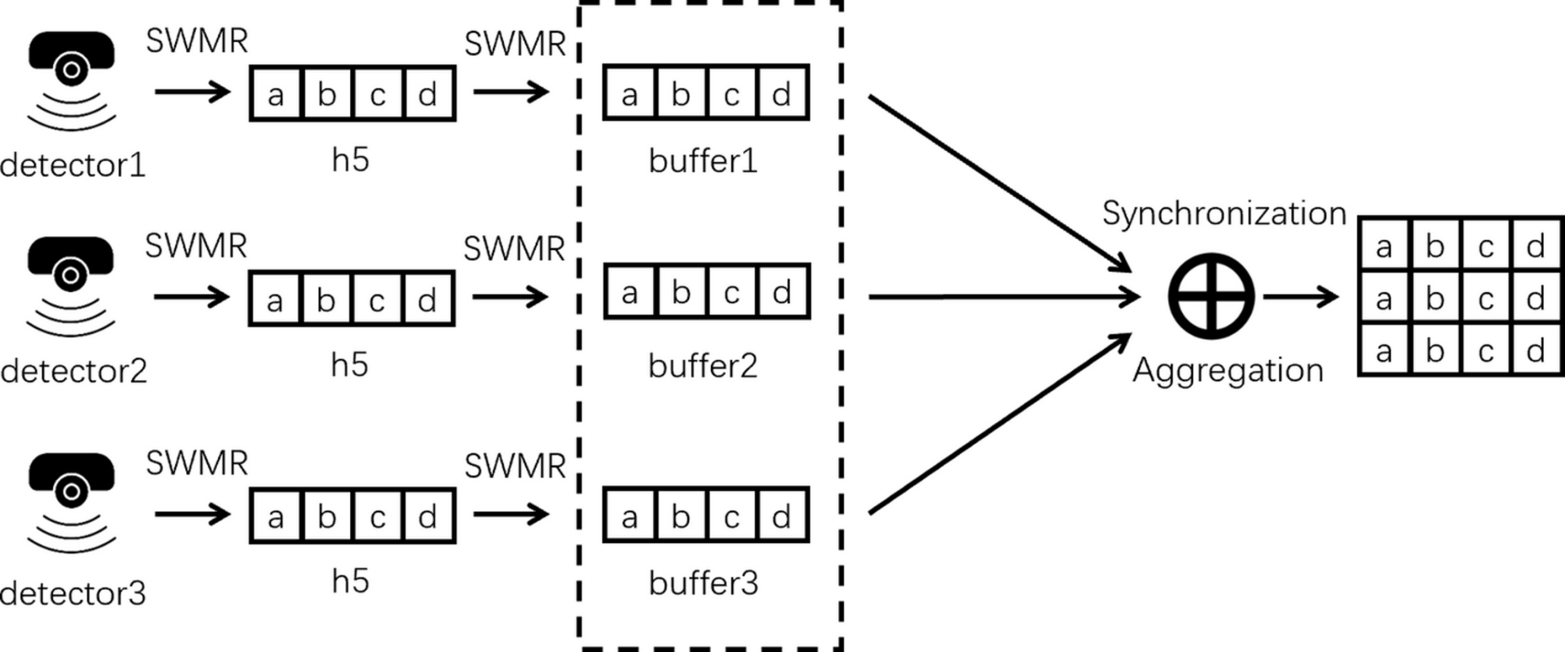
Architecture of multi-target data acquisition and frame-level alignment in the *MDW* pipeline, showing Detector Workers, the Align process with stream-map buffering, and merged output.

Once all files have accumulated at least length frames in their respective buffers, the system updates the global read index and proceeds to batch-pack the aligned data. Before packing, a temporal alignment step is performed: the frame numbers recorded by each detector device during acquisition are compared, and only data belonging to the same time window or sharing the same frame number are grouped together for synchronous encapsulation. This ensures that the output data packet faithfully represents a consistent snapshot across all detector sources, even when individual devices exhibit slight jitter in their acquisition timing. The packed multi-source data block is then transmitted to downstream systems—including storage workers, visualization clients, and computation center interfaces—via ZeroMQ messaging (Hintjens, 2013 ▸), as illustrated in Fig. 6 ▸.

### 4.3. Fluorescent target data storage

**Figure 7:**
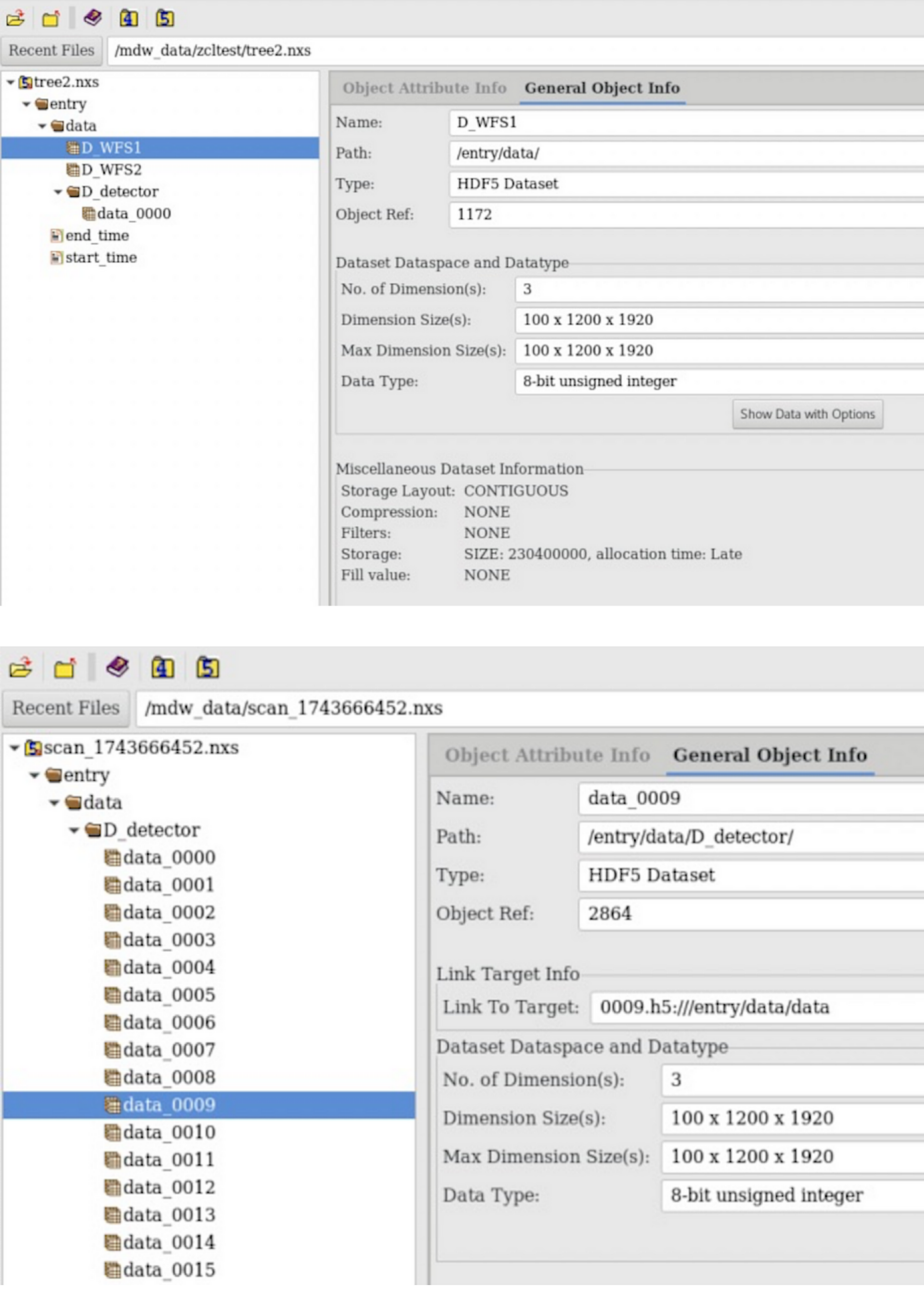
HDF5 storage dataset structure of the *MDW* Storage Worker.

Persistent storage of fluorescent target data is managed by the *MDW* Storage Worker, which writes acquired images and associated metadata into HDF5 files following a hierarchical dataset structure. The storage configuration is defined in YAML files specific to each beamline, specifying the mapping between incoming data fields and HDF5 dataset paths. A typical fluorescent-target dataset includes image arrays stored under /entry/data/data, motor positions under /entry/instrument/positioners/, scan timestamps under /entry/start_time and /entry/end_time, and scan identifiers under /entry/scan_id. To accommodate multi-modal data from heterogeneous detector sources, the *MDW* storage system organizes acquired datasets using a fluorescent-target-centric naming convention within the HDF5 file hierarchy. Each fluorescent target or diagnostic device is assigned a dedicated dataset under the /entry/data/ group, with the dataset name directly reflecting the device identifier. For example, as illustrated in the top panel of Fig. 7 ▸, image data from the first wavefront sensor are stored at the path /entry/data/D_WFS1, while data from the second wavefront sensor are written to /entry/data/D_WFS2. This naming scheme ensures intuitive data organization and enables downstream analysis tools to unambiguously associate stored arrays with their originating instruments, even when a single scan involves simultaneous acquisition from dozens of heterogeneous sources. For high-throughput acquisition scenarios—where individual datasets may grow to tens of gigabytes per scan—the storage engine further partitions each dataset into multiple fixed-size sub-chunks along the frame dimension. This chunked storage strategy offers several advantages: it bounds the memory required for each write operation, enables efficient partial reads without loading the entire dataset, and improves HDF5 internal B-tree indexing performance for large append-heavy workloads. The chunk size is configurable on a per-beamline basis through the YAML storage configuration, allowing operators to balance write granularity against I/O overhead according to the specific data rate and file system characteristics of each beamline station.

Upon scan completion, the Storage Worker also transmits metadata to the HEPS computation center and Apache Kafka (Garg, 2013 ▸), including the beam time identifier, scan identifier, sample name, file paths, and acquisition timestamps. This metadata submission enables automated cataloging and triggers downstream analysis pipelines. The combination of SWMR-enabled real-time access, structured HDF5 archival, and automated metadata distribution provides a complete data lifecycle management solution for fluorescent target imaging at HEPS beamlines.

### 4.4. HEPS beamline alignment database

**Figure 8:**
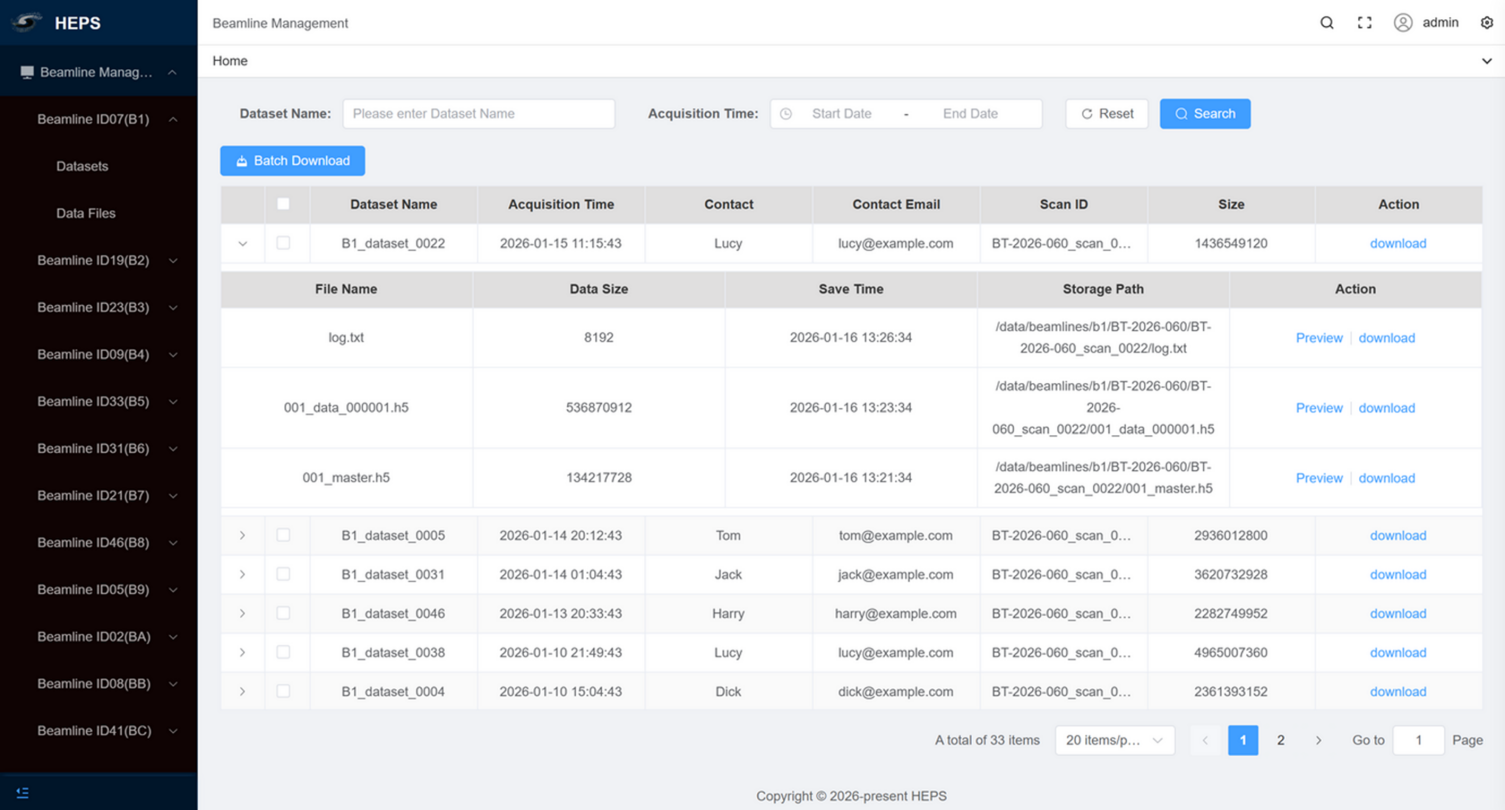
Beamline alignment database.

To support data analysis and model development for beamline alignment at HEPS, a dedicated beamline alignment database has been established based on the data orchestration framework of *MDW*. During fluorescent-target experiments, the corresponding fluorescent target data, together with key experimental information, are systematically organized and stored in this database (see Fig. 8 ▸), forming a specialized dataset for beamline alignment studies.

In the *MDW* architecture, experimental data generated by detectors are streamed to the *MDW* through high-throughput communication protocols based on ZeroMQ (Li *et al.*, 2023 ▸), enabling real-time data transfer and flexible pipeline scheduling. Within this framework, ZeroMQ is primarily used for efficient intra-system communication due to its low-latency and high-throughput characteristics. In addition, Apache Kafka is employed for asynchronous metadata dissemination, allowing experimental metadata (*e.g.* beam time identifiers, scan identifiers, and data file paths) to be distributed to downstream systems.

During data acquisition, raw detector data are written to HDF5 files via the *MDW* storage pipeline, while associated experimental metadata are published to Kafka topics and subsequently consumed by the beamline alignment database. The alignment database is implemented using a Django REST Framework (DRF) backend, which provides standardized JSON-based RESTful APIs for metadata ingestion, management, and querying. The system follows a client–server architecture, where the database service is containerized and deployed on beamline servers using Docker, and the HDF5 data files are accessed through shared storage.

The alignment database is implemented using a relational schema (MySQL), in which beam-time-level information (*e.g.* beam time ID, proposal ID, principal investigator, and experiment type) is linked to dataset-level metadata (*e.g.* scan ID, acquisition time, and contact information), and further associated with file-level records (*e.g.* file name, size, storage path, and save time). The HDF5 data files are stored on a shared network file system (*e.g.* NFS-mounted storage), enabling efficient access by downstream processing and visualization services.

The database is currently being extended with integrated modules for data processing and data cleansing, enabling standardized data organization and quality control. In addition, the construction of curated datasets for machine-learning-based beamline alignment is currently under development, with the aim of supporting future intelligent alignment strategies (Morris *et al.*, 2024 ▸). Overall, the alignment database provides a structured and reliable data foundation for beamline data management and future intelligent optimization at HEPS.

## 5. Conclusion

In this study, an integrated fluorescent-target imaging and real-time beam diagnostic platform was developed and deployed at HEPS. The system combines a radiation-tolerant, UHV-compatible mechanical assembly using diamond and YAG:Ce scintillators with a synchronized distributed multi-camera architecture implemented within the *EPICSareaDetector* framework. Experimental validation demonstrated stable operation and reliable quantitative performance under high-brightness beamline conditions. The modular motion system achieved a positioning accuracy of ±1 µm and a repeatability of ±5 µm, ensuring reproducible target deployment. The imaging architecture supported synchronized high-throughput acquisition from up to 13 distributed cameras without packet loss. Real-time image processing enabled IOC-side parameter extraction in less than 20 ms and an end-to-end acquisition-to-analysis latency below 50 ms, providing precise and temporally consistent beam characterization. Integration of the *MDW* framework further enabled multi-target data alignment, high-throughput distribution and persistent HDF5 storage, while diagnostic datasets and metadata were automatically organized into a dedicated beamline alignment database. Overall, this platform provides a scalable and reliable framework for beamline alignment, online diagnostics and coordinated data management at HEPS, and supports future data-driven optimization in fourth-generation synchrotron radiation facilities.
